# Determining minimal inhibitory concentrations and antibiotic susceptibility for Enterobacterales by flow cytometry using reactive oxygen species as a marker

**DOI:** 10.1371/journal.pone.0331217

**Published:** 2025-09-04

**Authors:** Jia Hao Yeo, Nasren Begam, Wan Ting Leow, Andrea Lay-Hoon Kwa

**Affiliations:** 1 Division of Pharmacy, Singapore General Hospital, Singapore; 2 SingHealth-Duke-NUS Academic Clinical Programme, Singapore; 3 Department of Pharmacy, National University of Singapore, Singapore; 4 Emerging Infection Diseases Program, Duke-NUS Graduate Medical School, Singapore; Yamagata University Faculty of Medicine: Yamagata Daigaku Igakubu Daigakuin Igakukei Kenkyuka, JAPAN

## Abstract

Early appropriate antibiotic treatment is vital in reducing patient mortality. However, current antimicrobial susceptibility testing (AST) requires 16–24 hours of incubation, delaying appropriate antibiotic treatment. Flow cytometry (FCM) is a rapid method in assessing fluorescence (such as fluorophores for ROS) at single-cell resolution. Reactive oxygen species (ROS) are oxygen-containing molecules, which are inducible by antibiotics and potentially bactericidal. We asked if FCM measurements of ROS in antibiotic-treated bacteria could be used in determining antibiotic MIC as an alternative to conventional AST. This study aims to develop and evaluate the feasibility of a FCM assay to determine antibiotic susceptibility accurately with a short turn-around time. MICs of amikacins, aztreonams, cephalosporins (with or without a lactamase inhibitor), carbapenems, levofloxacin, polymyxin B, trimethoprim/sulfamethoxazole, and tigecycline were determined for six clinical carbapenem-resistant Enterobacterales isolates using conventional microbroth dilution assays and using FCM assessments in parallel. Accurate MICs determined using FCM is defined as MICs falling within 2-fold dilutions of the conventional microbroth dilution AST assay. MIC determination via ROS measurements were mostly accurate for carbapenems (22/24; 91.7% accuracy) and trimethoprim/sulfamethoxazole (5/6; 83.3% accuracy). In contrary, ROS levels were less accurate in determining MICs for amikacin (4/6; 66.7% accuracy), aztreonam (4/6; 66.7% accuracy), cephalosporins only (5/12; 41.6% accuracy), cephalosporin with lactamase inhibitor (11/18; 61.1% accuracy), polymyxin-B (2/6; 33.3% accuracy), levofloxacin (1/6; 16.7% accuracy), and tigecycline (2/6; 33.3% accuracy). These data support that ROS assessments using FCM is suitable for accurately determining MICs for carbapenems in Enterobacterales. Further optimisation and validation of this FCM assay with additional bacteria strains with varying antibiotic susceptibilities are warranted. Future studies include assessing other organisms and antibiotic pairs.

## Introduction

Carbapenem-resistant Enterobacterales (CRE) is a major public health threat globally and is associated with increased mortality, length of hospitalisation stay, and hospitalisation costs [1,2]. Carbapenem-resistant *Klebsiella pneumoniae* and carbapenem-resistant *Escherichia coli* account for more than 90% of the CRE strains widely disseminated worldwide through multiple routes [3,4].

In Singapore, clinical CRE is trending among adult inpatients since 2010 [5–7]. A study conducted at the largest tertiary hospital in Singapore revealed carbapenem resistance rates to increase more than five-folds within four years (between 2011 and 2015), with *Klebsiella pneumoniae* (from 2% to 12%) and *Escherichia coli* (from 0% to 5%) [5]. A 2017 study reviewing on Hospital Acquired Infections in Singapore revealed that 7% of all Enterobacterales implicated in Hospital Acquired Infections, are resistant to carbapenems [6,7].

Timely selection of appropriate antibiotics for effective treatment of CRE infections is essential to ensure survival [8]. Current antibiotic susceptibility testing (AST) methods require incubating bacteria with antibiotics for 18–24 hours. Hence, a shorter turnaround time for AST is highly desired.

Exposing bacteria to sufficient antibiotics can increase reactive oxygen species (ROS) causing oxidative damage, which are potentially bactericidal [9–13]. Fluorescence methodologies, such as flow cytometry (FCM), remain one of the simplest measurements of ROS in live cells [14–16]. Previously, we showed high accuracies in determining MICs of β-lactams in *Acinetobacter baumannii* by FCM using ROS as a marker [17]. Here, we extend our studies further in Enterobacterales. We hypothesized that FCM assessments of ROS can also accurately determine antibiotic susceptibilities in Enterobacterales. We aim to develop and evaluate the feasibility of a rapid high-throughput FCM AST assay that only requires a short turnaround time.

## Materials and methods

### Bacteria isolates

Nonclonal clinical strains of carbapenem-resistant Enterobacterales previously collected from the largest tertiary hospital in Singapore as part of a nationwide surveillance study from 2013 to 2016, were used in this study. Genus identities were determined using Vitek GNI1 cards with the Vitek 2 instrument (bioMérieux, Hazelwood, MO, USA) and/or a matrix-assisted laser desorption/ionization time-of-flight mass spectrometry (MALDI-TOF MS) system (Bruker Daltonik, Germany) as part of the routine workflow of the microbiology laboratory at the institution. Bacteria isolates were stored at −80^0^C. Isolates were sub-cultured twice on Trypticase Soy Agar (TSA) with 5% sheep blood (Thermo Fisher, Singapore) at 35°C before each experiment. Isolates used in this study were selected based on the respective MICs of antibiotics (S1 Table). These isolates have MICs of all antibiotics within the range of antibiotic concentrations in the antibiotics panel.

### Fluorophores used for FCM

Fluorescent dye, RedoxSensor^TM^ Green (RSG) of the *BacLight* RedoxSensor Green Vitality Kit (Thermo Fisher Scientific, Singapore), was used to assess bacterial ROS. Increased RSG fluorescence intensity indicates increased intracellular ROS. To identify viable bacteria for ROS assessments, the fluorescent dyes, SYTO-62 and propidium iodide (PI), (Thermo Fisher Scientific, Singapore) were used together with RSG. SYTO-62 labels nucleic acid of all bacteria. PI only enters non-viable bacteria with compromised membranes and intercalate DNA.

### Multi-color FCM assessments

Bacterial suspension of 10^7.7^ CFU/mL in physiological saline (0.85% (w/v) sodium chloride) was prepared. Ten-fold dilutions were performed to achieve 10^5.7^ CFU/mL in cation-adjusted Müller-Hinton broth. Microtiter broth dilution panel was inoculated with 100 µL of bacterial suspension at 10^5.7^ CFU/mL per well. Bacteria suspension at 10^5.7^ CFU/mL was inoculated into each well of a customized microtiter broth dilution panel plate. Plate was incubated at 35°C for 60 minutes. Bacteria were then stained with the fluorescence cocktail (20 µM RSG, 1 µM SYTO-62 and 20 µM PI) and incubated for a further 15 minutes at room temperature before FCM assessments. [17] Data acquisition was carried out with CytoFlex flow cytometer (Beckman Coulter, Singapore). Details on instrumental settings and configurations during data acquisition can be found in S2 Table. Manufacturer settings with a default “medium” flow rate of 30 µL/min was used. Each well was sampled for 180 seconds or until 20,000 events were collected, whichever came first. Durations were set at 5 s and 60 s for sample mixing and backflush of the fluidic system respectively to ensure accuracy of FCM assessments. Individual wells of the customized antibiotic panel were assessed randomly for each run and for each organism. Data acquired were exported in.fcs files and analyzed using the FlowJo software (v10.4, Tree Star, Ashland, OR, USA). Gating strategy and compensation matrix are shown in S1 Fig and S3 Table respectively.

### Standard microtiter broth dilution method

MIC was determined using standard microtiter broth dilution method with customized 96-well microtiter broth dilution panels (Trek Diagnostics, East Grinstead, UK) performed under CLSI guidelines. Briefly, bacterial suspension of 10^7.7^ CFU/mL in physiological saline (0.85% (w/v) sodium chloride) was prepared. Ten-fold dilutions were performed to achieve 10^5.7^ CFU/mL in cation-adjusted Müller-Hinton broth. Microtiter broth dilution panel was inoculated with 100 µL of bacterial suspension at 10^5.7^ CFU/mL per well. Bacterial growth is assessed after the microtiter plate was then incubated at 35°C over 20–24 hours. Susceptibility breakpoints from MICs were interpreted primarily based on CLSI guidelines.

### Statistical comparisons between methodologies

Percentages were used as a descriptive statistic to determine accuracies rudimentarily of the MICs determined by FCM as compared to the standard microtiter broth dilution. Bland-Altman analyses were used to assess agreement of MICs determined by FCM and microbroth dilution methods (S1 File). MICs were log₂-transformed prior to comparison. For MICs ≥ 32 µg/mL, the next larger 2-fold value (i.e., 64 µg/mL) were used for logarithmic transformation. Mean difference (đ) and 95% limits of agreement (± 1.96 × SD), with the standard errors were computed and visualized.[18] Percent differences were calculated as [(log₂(MIC_FLOW_) − log₂(MIC_BROTH_))/ mean] × 100%.[19] To determine for proportional bias, a linear regression between the percentage difference and the average of the two methods was performed. A *P*-value < 0.05 was considered statistically significant. A statistically significant non-zero slope would indicate proportional bias.

## Results

### Determining optimal time in incubation with antibiotics for assessing ROS as a marker by flow cytometry (FCM)

Alterations in metabolism associated such as oxidative damage are present as short as 30 minutes of antibiotics exposure [20]. On the other hand, inaccuracies can arise from a longer duration required to generate ROS by certain antibiotics [21]. Hence, we first performed pilot studies to optimize the shortest possible time required for the largest RSG fluorescence measurements possible, without massively affecting the viability or structural integrity of bacteria from ROS damage.

We investigated ROS as a marker in our assay to predict MIC for Enterobacterales. Using a cocktail of various fluorophores, we assessed oxidative stress in viable bacteria after exposure to antibiotics. This fluorophore cocktail consists of RedoxSensor^TM^ Green (RSG), SYTO-62 and propidium iodide (PI). Viable bacteria are defined as bacteria stained with SYTO-62 but not PI (SYTO-62^POSITIVE^PI^NEGATIVE^). RSG senses the intracellular redox environment in bacteria by labeling ROS. An increase in intracellular ROS will result in an increase in RSG fluorescence intensity measurable by flow cytometry. Our previous study has established that RSG can sense hydrogen peroxide (H_2_O_2_) and hypochlorite anion (HOCl^-^) in bacteria. [17]

**Fig 1:**
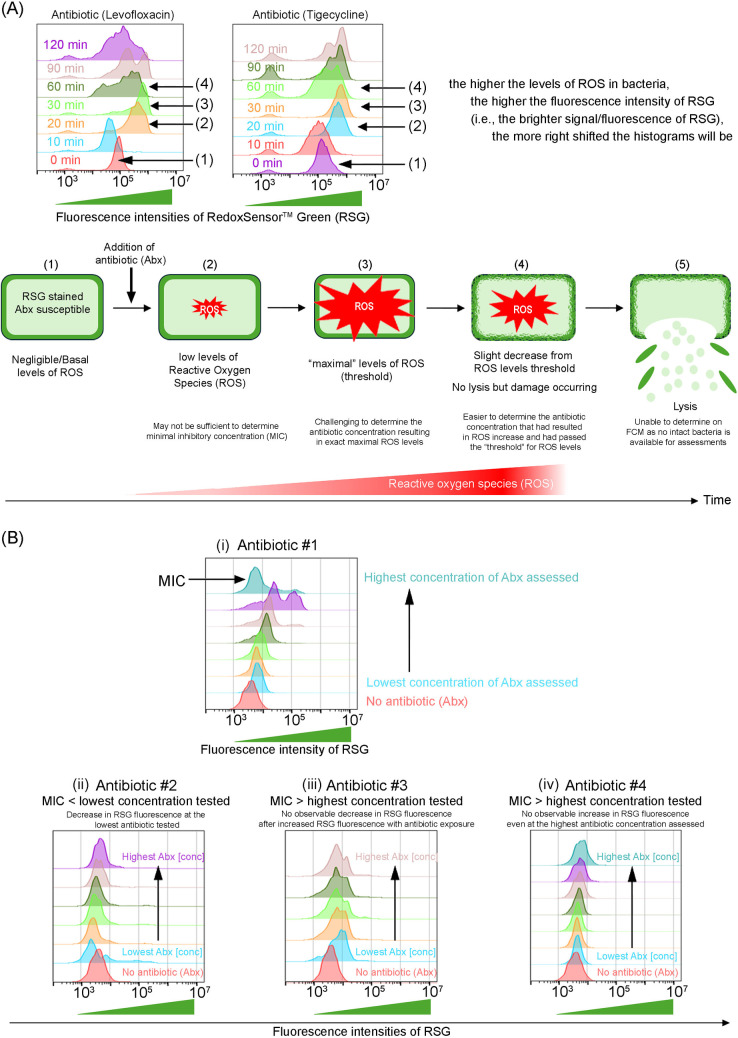
Antibiotics increase ROS in antibiotic-susceptible bacteria. (A) Sequence of intracellular redox events after antibiotic exposure over a short duration. Clinical isolate susceptible to levofloxacin (MIC < 20 mg/L) and tigecycline (MIC < 2 mg/L) was used to determine the optimal antibiotic exposure time. Antibiotic concentrations used were clinical achievable concentrations obtained from literature [22,23]. Representative FCM plots revealing RSG fluorescence at each time interval post-antibiotic exposure were shown. Sequence of events post-antibiotic exposure (1 to 5) inferred from the FCM plots were depicted in the schematic and described in the main text. (B) The MIC from our FCM assay using ROS as a marker was then determined using the sequence of intracellular redox events after antibiotic exposure determined in the above panel (A). (i) The MIC concentration of any antibiotic is determined to be the lowest concentration revealing a reduction in RSG fluorescence after an increase in RSG fluorescence at even lower antibiotic concentrations. (ii) In FCM plots showing a decrease in the lowest antibiotic concentration tested, the MIC is determined to be lower than the lowest concentration tested (i.e., MIC < lowest concentration tested). (iii) FCM plots revealing no observable decrease in ROS levels after an increase of RSG fluorescence was observed at lower antibiotic concentrations, the MIC is determined to be higher than the highest concentration tested (i.e., MIC > highest concentration tested). (iv) When histograms show no shift at RSG fluorescence, the MIC is determined to be higher than the highest concentration tested (i.e., MIC > highest concentration tested).

Antibiotic-susceptible bacteria were first exposed to antibiotics (Fig 1A, panel 1). At each time interval, bacteria were stained further with the fluorophores prior to FCM assessments. The same gating strategy in our previous studies was adopted [17].

Approximately 20 mins after antibiotic exposure, there was an increase in bacterial ROS (Fig 1A, panel 2). At approximately 30 mins post-antibiotic exposure, maximal rightward shift of RSG fluorescence compared to 0 min in FCM plots was observed, indicating maximal levels of ROS (deemed as threshold; Fig 1A, panel 3). No significant impact on the MIC readout for extended periods of incubation time at room temperature while waiting for acquisition.

While this is ideal for assessing MIC using ROS as a marker, ROS are temporally transient, hence, difficult to determine if the increase in RSG fluorescence is at its maximal (“threshold”). After the accumulation of ROS to a “threshold”, diminished RSG fluorescence was observed indicating a slight decrease in ROS (Fig 1A, panel 4). Further incubation with antibiotic will result in more bacterial lysis (Fig 1A, panel 5), which will be impossible to define the MICs using FCM, as the fluorophores only labels intact bacteria. Hence, these optimization studies determined 60 minutes is required to ensure optimal duration to assess ROS in Enterobacterales across all antibiotics.

### Obtaining MICs using multi-color FCM across 15 different antibiotics

We next investigated ROS as a marker in our assay to predict MIC. Six clinical isolates of Enterobacterales (three *E. coli*, three *K. pneumoniae*) were incubated with varying concentrations of antibiotics in a customized 96-well microtiter plate for 60 minutes. These antibiotics include amikacin, aztreonam, ceftolozane/tazobactam, ceftaroline, ceftazidime/avibactam, doripenem, ertapenem, cefepime, imipenem, levofloxacin, meropenem, polymyxin B, trimethoprim/sulfamethoxazole (Bactrim), tigecycline, and piperacillin/tazobactam. Without removing the antibiotics, bacteria were then stained with the fluorescence cocktail for a further 15 minutes at room temperature. This fluorophore cocktail consists of RSG, SYTO-62 and propidium iodide (PI). Viable bacteria are defined as bacteria stained with SYTO-62 but not PI.

**Fig 2:**
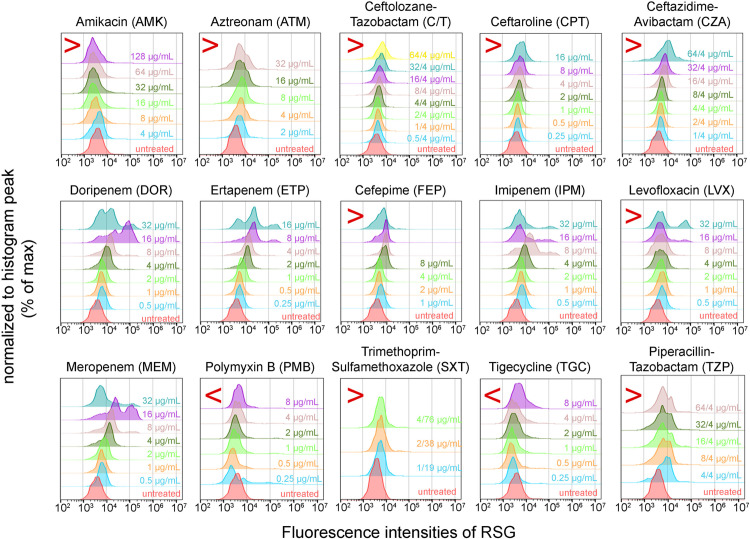
Assessing MICs in clinical isolated Enterobacterales via FCM using ROS as a marker. (A) Representative FCM plots showing RSG fluorescence intensities assessed in clinical isolate (*Klebsiella pneumoniae*) after exposing to 15 different antibiotics at varying concentrations for 60 mins. Rightward shifts of histograms indicate increased RSG fluorescence, which implies increased bacterial ROS. MICs determined by FCM were indicated in red circle for each antibiotic. The ‘>’ symbol indicates MIC is higher than the highest concentration of antibiotic assessed, while ‘<’ symbol indicates MIC is lower than the lowest concentration of antibiotic assessed.

MICs of these antibiotics determined by FCM was defined as the first antibiotic concentration to reveal a substantial decrease in RSG fluorescence intensity immediately after an increasing RSG fluorescence intensity with increasing antibiotic concentrations (Fig 1B, panels i – iv). This ensures that ROS accumulation have passed its threshold in the bacteria. Representative flow cytometric data with MICs determined (marked in red) for *Enterobacteriaceae* clinical isolate, *Klebsiella pneumoniae* (ID:EC0997) is shown in Fig 2. These MICs obtained were verified against the MICs obtained via microtiter MIC method under CLSI guidelines [24].

### Comparing the MICs obtained from FCM AST against MICs obtained from standard microtiter broth dilution method

We then determined the accuracy of our FCM-AST assay by comparing the MICs obtained using flow cytometry, to the MICs obtained using conventional microbroth dilution AST assay. Accurate MICs is defined as MICs falling within 2-fold dilutions of the conventional microbroth dilution AST assay that runs simultaneously, performed under CLSI guidelines.

Determining MICs by FCM were mostly accurate for carbapenems (91.7% accuracy) and trimethoprim/sulfamethoxazole (83.3% accuracy) (Table 1). In contrary, ROS levels were less accurate in determining MICs for amikacin (50% accuracy), aztreonam (66.7% accuracy), cephalosporins only (41.6% accuracy), cephalosporins with lactamase inhibitor (61.1% accuracy), levofloxacin (16.7% accuracy), polymyxin B (33.3% accuracy), and tigecycline (33.3% accuracy). As MICs for Bactrim (SXT) exceeded the measurable range in our panel as indicated by the “≤” and “≥” symbols, we performed statistical assessments only on the carbapenem MICs to compare the two methodologies.

**Table 1:** MICs and antibiotic susceptibilities obtained from conventional microbroth dilution and flow cytometry methodologies.

Organism		*Klebsiella Pneumoniae*						*Escherichia coli*						Total hits (%)
Isolate		EC0329		EC0997		EC1751		EC0238		EC0381		EC0644		
ABx classification	ABx (mg/L)	MIC_BROTH_	MIC_FLOW_	MIC_BROTH_	MIC_FLOW_	MIC_BROTH_	MIC_FLOW_	MIC_BROTH_	MIC_FLOW_	MIC_BROTH_	MIC_FLOW_	MIC_BROTH_	MIC_FLOW_	
Amikacin	AMK	**≤4 (S)**	**≤4 (S)**	≤4 (S)	≥256 (R)	**≤4 (S)**	**≤4 (S)**	≤4 (S)	≥256 (R)	**≤4 (S)**	**≤4 (S)**	**8 (I)**	**≤4 (S)**	4/6 (66.7%)
Aztreonam	ATM	≥64 (R)	32 (R)	**≥64 (R)**	**≥64 (R)**	**16 (R)**	**16 (R)**	**≥64 (R)**	**≥64 (R)**	**≥64 (R)**	**≥64 (R)**	8 (I)	≤2 (S)	4/6 (66.7%)
Bactrim	SXT	≤1/19 (S)	≥8/152 (R)	**≥8/152 (R)**	**≥8/152 (R)**	**≤1/19 (S)**	**≤1/19 (S)**	**≥8/152 (R)**	**≥8/152 (R)**	**≤1/19 (S)**	**≤1/19 (S)**	**≥8/152 (R)**	**≥8/152 (R)**	5/6 (83.3%)
Carbapenems	DOR	**4 (R)**	**4 (R)**	**32 (R)**	**32 (R)**	1 (S)	8 (R)	**4 (R)**	**4 (R)**	**2 (I)**	**2 (I)**	**4 (R)**	**8 (R)**	22/24 (91.67%)
	ETP	16 (R)	≥32 (R)	**16 (R)**	**16 (R)**	**4 (R)**	**4 (R)**	**16 (R)**	**16 (R)**	**4 (R)**	**8 (R)**	**4 (R)**	**8 (R)**	
	IPM	**16 (R)**	**16 (R)**	**16 (R)**	**16 (R)**	**8 (R)**	**8 (R)**	**8 (R)**	**8 (R)**	**8 (R)**	**8 (R)**	**8 (R)**	**8 (R)**	
	MEM	**8 (R)**	**16 (R)**	**32 (R)**	**32 (R)**	**2 (I)**	**2 (I)**	**8 (R)**	**8 (R)**	**4 (R)**	**4 (R)**	**8 (R)**	**16 (R)**	
Cephalosporin only	CPT	**≥32 (R)**	**16 (R)**	**≥32 (R)**	**≥32 (R)**	≥32 (R)	8 (R)	≥32 (R)	4 (R)	**≥32 (R)**	**≥32 (R)**	**≥32 (R)**	**16 (R)**	5/12 (41.7%)
	FEP	4 (I)	32 (R)	32 (R)	≥128 (R)	2 (S)	32 (R)	≥128 (R)	32 (R)	**64 (R)**	**≥128 (R)**	2 (S)	32 (R)	
Cephalosporin + β-lactamase inhibitor	C/T	**8/4 (R)**	**4/4 (I)**	**≥128/4 (R)**	**≥128/4 (R)**	**4/4 (I)**	**4/4 (I)**	**16/4 (R)**	**32/4 (R)**	**≥128/4 (R)**	**≥128/4 (R)**	2/4 (S)	32/4 (R)	11/18 (61.1%)
	CZA	**≤1/4 (S)**	**≤1/4 (S)**	**≥128/4 (R)**	**≥128/4 (R)**	**≤1/4 (S)**	**≤1/4 (S)**	2/4 (S)	16/4 (R)	**≤1/4 (S)**	**≤1/4 (S)**	≤1/4 (S)	64/4 (R)	
	TZP	≥128/4 (R)	16/4 (I)	32/4 (R)	≥128/4 (R)	≥128/4 (R)	16/4 (I)	**≥128/4 (R)**	**≥128/4 (R)**	**≥128/4 (R)**	**≥128/4 (R)**	≥128/4 (R)	16/4 (I)	
Levofloxacin	LVX	≤0.5 (S)	8 (R)	≤0.5 (S)	≥64 (R)	≤0.5 (S)	16 (R)	1 (I)	4 (R)	≤0.5 (S)	≥64 (R)	**16 (R)**	**16 (R)**	1/6 (16.7%)
Polymyxin B	PMB	0.5 (S)	≤0.25 (S)	0.5 (S)	≤0.25 (S)	**≤0.25 (S)**	**≤0.25 (S)**	≤0.25 (S)	0.25 (S)	1 (S)	≤0.25 (S)	**2 (R)**	**4 (R)**	2/6 (33.3%)
Tigecycline	TGC	**≤0.25 (S)**	**≤0.25 (S)**	0.5 (S)	≤0.25 (S)	**2 (S)**	**1 (S)**	≤0.25 (S)	≥16 (R)	≤0.25 (S)	≥16 (R)	0.5 (S)	≤0.25 (S)	2/6 (33.3%)
**Inoculum (Log**_**10**_ **CFU/mL)**		5.64	5.83	5.68	5.78	5.03	5.03	5.82	6.39	5.93	5.90	5.83	5.82	

Table summarizing MICs of antibiotics obtained from both conventional microbroth dilution assay and flow cytometry for both *Escherichia coli* and *Klebsiella pneumoniae*. Susceptibility breakpoints interpreted from MICs were based on CLSI guidelines. MICs for respective antibiotic as determined by both techniques, found within 2-fold dilution are deemed as “accurate/hit” and are indicated in **bold**. Abbreviations in Table - ABx: Antibiotics; AMK: Amikacin; ATM: Aztreonam; C/T: Ceftolozane/Tazobactam; CPT: Ceftaroline; CZA: Ceftazidime/Avibactam; DOR: Doripenem; ETP: Ertapenem; FEP: Cefepime; IPM: Imipenem; LVX: Levofloxacin; MEM: Meropenem; MIC_BROTH_: Minimal inhibitory concentration obtained from conventional microbroth dilution assay; MIC_FLOW_: Minimal inhibitory concentration obtained from flow cytometric assessments of reactive oxygen species; PMB: polymyxin B; SXT: Trimethoprim/Sulfamethoxazole (Bactrim); TGC: Tigecycline; TZP: Piperacillin/Tazobactam

**Fig 3:**
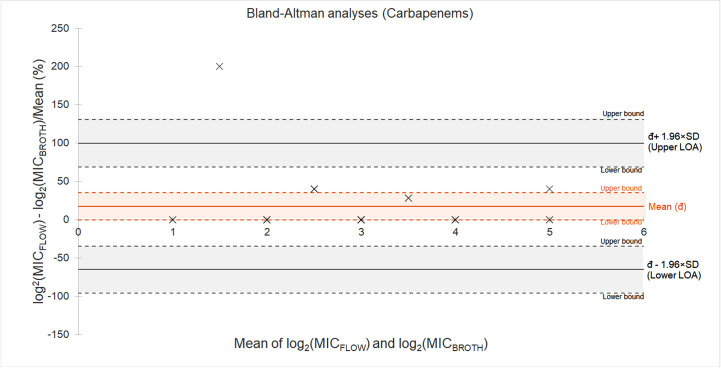
Both methodologies are broadly comparable when assessing carbapenems. Bland-Altman analysis was performed to describe and compare the agreement of MICs from FCM and microtiter broth methods. A Bland-Altman plot was shown to depict the analysis. The y-axis shows the percent difference: [(MIC_FLOW_ − MIC_BROTH_)/ mean] × 100%. The x-axis shows the mean MICs between the two methods. Solid orange line indicates the mean difference (bias), and the solid gray lines represent the 95% limits of agreement (mean (đ) ± 1.96 × SD). Dotted lines represent the upper and limit bounds, while shaded regions indicate areas covered by the upper and lower bounds, for means, upper LOA and lower LOA.

A Bland–Altman analysis was conducted to evaluate the agreement between MIC values measured by FCM and the standard broth microdilution method for carbapenems (Fig 3). [18,19] The differences were expressed as percentage bias relative to the average of the two methods. The mean bias was 17.4%, indicating that MICs obtained from FCM were slightly higher than MICs obtained from microtiter broth dilution. The 95% limits of agreement (LOA) ranged from –65.2% to 99.9%, suggesting substantial variability between methods in individual cases. The confidence interval (CI) for the mean bias spanned from –0.4% to 35.2%. The CI for the lower LOA ranged from –95.97% to –34.33%, while the CI for the upper LOA ranged from 69.09% to 130.73%. These results suggest a moderate systematic bias with wide limits of agreement. This implies that the methods are broadly comparable but further standardization is required for the methods to be interchangeable.

Linear regression comparing between the percent difference and the average MIC values indicates that the differences were evenly distributed around the mean across the range of MICs, with no evidence of proportional bias (β/slope = –8.85, 95% CI: –24.90 to 7.20, *P* = 0.264) (S1 File). The coefficient of determination (R^2^ = 0.059) indicated a weak and non-significant relationship between average MIC and measurement difference. Therefore, linear regression indicates that the sole use of Bland–Altman analysis for agreement, without the need to adjust for systematic proportional error.

## Discussion

Timely selection of appropriate antibiotics for effective treatment of CRE infections is essential to ensure survival [8,25]. Current AST methods involve using culture-based methods, such as broth dilution and agar dilution assays. These culture methods are still gold standards with high levels of sensitivity and specificity. However, current AST methods require 16–24 hours incubation following isolation of pure bacterial colonies [26,27]. This delays administration of appropriate antibiotics thereby increasing the likelihood of infection-related mortality. While rapid AST methods such as molecular and mass spectrometry-based methods were proposed and have been compared against the gold standards, these rapid AST methods do not generally specify the exact minimal inhibitory concentration or the antibiotic susceptibility [28].

Flow cytometry (FCM) has been considered for rapid AST since the 1980s. In this assay, time lapsed for the instrument to assess the entire customized antibiotic panel plate was approximately 4–6 h per run, as compared to the gold standard that requires overnight incubation time. Minimal inhibitory concentrations of any antibiotic is a susceptibility phenotypic expression of that specific bacteria [29,30]. Any time elapsed between the first and last well assessed, containing the same antibiotic concentration, will not affect the result.

As we have discussed earlier [17], FCM triumphs in speed, i.e., rapid turnaround time, and the ability to assess single cells as compared to classical incubation methodologies [31–33]. Our optimized setup would exclude cellular debris or mammalian cells, increasing the sensitivity of our assay. FCM assays are also shows good correlation in accuracy and offers higher flexibility compared to current automated AST tools, such as Vitek-2 [34–38]. One example would be in the event of a polymicrobial infection, we envisioned the workflow to isolate and identify individual clinically relevant pathogen via microbiological cultures according to CLSI guidelines and establish an antibiotic panel for AST testing using our FCM AST assay setup.

However, we are unable to ignore the drawbacks stemming from technical expertise required for data analyses and high variability in data analyses between technical personnels. To improve reproducibility and reduce user variability, we adopted auto-contouring gating [17]. We envisioned an automated workflow from the extractions of mode fluorescence intensities and subsequent comparison to a single MIC value output without human intervention [39].

We reasoned that the inaccuracies in our assay stemmed from the different mechanisms of actions of individual antibiotics. Polymyxin B rapidly binds and neutralizes the lipopolysaccharide of Gram-negative bacteria, disrupting membrane integrity. Due to the rapid pharmacokinetics of polymyxin-B, 60 minutes might not be the optimal duration to assess viable cells for polymyxin-B induced oxidative stress [40]. Aztreonam is associated with oxidative stress induced by Fenton reaction [41], with the resultant hydroxyl radicals from Fenton reaction being difficult to be assessed by RSG [17]. Retrospective studies via surveying literature showed that ESBL-producing *E. coli* isolates were more susceptible to piperacillin-tazobactam (TZP) than ESBL-producing *Klebsiella* species *in vitro* [42]. This is similar to our observations with higher accuracies in predicting TZP susceptibility in *E. coli* than *K. pneumoniae*. We propound a theory that TZP may have different bactericidal mechanisms against *E. coli* and *K. pneumoniae*, as compared to other cephalosporin and beta-lactamases.

Reactive oxygen species (ROS) can be scavenged or quenched by antioxidants or enzymes, such as catalase. All three bacterial species assessed hitherto are generally known to possess catalase (i.e., catalase-positive), which reduces hydrogen peroxide (H_2_O_2_) to water (H_2_O). The RSG fluorophore in our FCM AST assay senses hydrogen peroxide in both obligate aerobes and facultative anaerobes post-antibiotic exposure. This suggests that β-lactams (including carbapenems) induce overwhelming levels of ROS that induces excessive oxidative stress in bacteria, observable by FCM.

Other than the differences in bacterial species assessed as compared to our previous study [17], we further categorized the β-lactams used in this study into cephalosporins only, cephalosporins with a β-lactamase inhibitor and carbapenems. The MICs of carbapenems determined using FCM attained a high accuracy of 91.7% against the classic broth microdilution assay. Due to logistical handicap, we are unable to assess carbapenem-susceptible strains and determine if this accuracy is attributed from the carbapenemases present in these carbapenem-resistant isolates.

Another limitation of this study lies in finding suitable isolates with MICs of antibiotics falling within the concentration range to be tested. Almost all carbapenem-resistant clinical isolates have very high MICs against most antibiotics (dilutions higher than the concentration assessed in this study). This renders most isolates not suitable for this study, hence the low sample numbers. Due to the limited sample size, we acknowledged the “proof-of-concept” nature of this study limits the ability to draw even broader conclusions and fully determine the performance of several antibiotic classes (such as cephalosporins and amikacins, which are approximately 50% accurate).

As mentioned our earlier study [17], we reiterate that our studies neither contest nor concur with associating ROS as the leading bactericidal cause of antibiotics as debated in literature [43–45]. Instead, this study revolved around exposing bacteria to a stress stimulus, such as sufficient antibiotic concentrations, resulting in an increase in bacterial ROS. In summary, this study described the workflow of a rapid FCM assay to determine MICs and antibiotic susceptibilities in Enterobacterales. Consistent to our earlier assessments on *Acinetobacter baumannii* [17], ROS assessments via FCM is suitable for determining MICs of carbapenems (which are also β-lactams) in Enterobacterales. The workflow can produce results within 6 hours, up to 19 hours earlier compared to standard AST methods. Our assay can potentially translate to faster initiation of appropriate antibiotics, and hence, improved clinical outcomes. Further studies will aim to optimize this setup in attaining our goal for an “one-protocol-fits-all-antibiotics” FCM-AST workflow for Enterobacterales, such as assessing and incorporating other biomarkers into the assay.

## Supporting information

S1 Table Genomic information of the clinical isolated bacteria strains used in this study. Overnight bacterial cultures in cation-adjusted Mϋller-Hinton broth were prepared and used for genomic DNA extraction using the DNeasy Blood and Tissue Kit (Qiagen GmbH, Hilden, Germany) according to the manufacturer’s instructions. Genomic DNA was then sent for paired-end WGS using MiSeq/HiSeq systems (Illumina Inc., CA, USA), with a resultant sequencing depth of at least 50-fold. Sequences were assessed for quality. Genomic data were further processed as summarised in this table. Acknowledgements to Dr Jocelyn Teo (Division of Pharmacy, Singapore General Hospital) and Dr Zhong Yang (Department of Clinical Translational Research, Singapore General Hospital) for their efforts in subsequent genomic characterization and bioinformatic analyses.
(XLSX)

S2 Table Instrumental settings on the CytoFlex flow cytometer. A CytoFlex® flow cytometer (Beckman Coulter, Brea, CA, USA) at the basic 4 + 3 + 2 configuration was used in this study. The flow cytometer is equipped with 405 nm, 488 nm and 640 nm lasers to excite fluorophores. A summary of fluorophores detected by corresponding detectors and gain voltages applied to the respective detectors were shown.
(DOCX)

S1 Fig Gating strategy used for analyzing flow cytometry data to assess redox status in viable bacteria. Schematic showing standardized gating strategy applied in flow cytometric data analyses. Gating identifies population of interest by placing a boundary (also known as gates) on flow cytometry plots. A gating strategy was implemented to identify redox status in viable bacteria. To minimize user-bias during flow cytometric analyses, the auto-contouring gate function on FlowJo analyses software was applied on contour plots at 2% threshold. These automated gates were placed to encompass as many concentric rings of contour as possible. A bacteria gate was first applied to identify bacteria using forward scatters (height) and side scatters (height). This is followed by a doublet discrimination gate to ensure only single cells are included for data analyses. Subsequently, a noise elimination gate was applied to reduce electronic noise for better accuracy and specificity. A viable bacteria gate was then placed for events that are SYTO-62^POSITIVE^Propidium-Iodide^NEGATIVE^. The viable bacteria gate is placed in reference to single stain controls to determine if populations are negative or positive for a marker/stain. Viable bacteria were then assessed for RSG fluorescence intensity (histogram). Histograms were then overlaid in a staggered manner, as presented throughout the manuscript.
(DOCX)

S3 Table Compensation matrix used for spectral spill-over of each fluorophore into respective detector used for FCM. Acquired flow cytometric data were analyzed using FlowJo. This table shows the matrix used for compensating against the spectral overlap and spillover using the FlowJo software.
(DOCX)

S1 File Details on the statistical analyses performed. Details of both the Bland-Altman and Linear Regression analyses (including raw data and equations) were shown in this document.
(DOCX)
