# Supplementary material for: Determining minimal inhibitory concentrations and antibiotic susceptibility for Enterobacterales by flow cytometry using reactive oxygen species as a marker
Source: PLoS One. 2025 Sep 4;20(9):e0331217. doi: 10.1371/journal.pone.0331217 (PMC12410812; doi:10.1371/journal.pone.0331217)
Supplement: S2 Table — A CytoFlex® flow cytometer (Beckman Coulter, Brea, CA, USA) at the basic 4 + 3 + 2 configuration was used in this study. The flow cytometer is equipped with 405 nm, 488 nm and 640 nm lasers to excite fluorophores. A summary of fluorophores detected by corresponding detectors and gain voltages applied to the respective detectors were shown. (DOCX) [file pone.0331217.s002.docx]

**S2 Table: instrumental settings on the CytoFlex flow cytometer**

| **Parameter** | **Assessments/Purpose** | **Detector Gain** |
| --- | --- | --- |
| Forward Scattering (FSC)-H | Size | 800 V |
| Side Scattering (SSC)-H | Granularity | 2800 V |
| FITC-H | RedoxSensor Green | 800 V |
| PC5.5-H | Propidium iodide | 150 V |
| APC-H | SYTO-62 | 800 V |
| PB450-H  (empty fluorescence channel 1) | Noise elimination | 51 V (default) |
| KO525-H (empty fluorescence channel 2) | Noise elimination | 27 V (default) |
