# Supplementary material for: Determining minimal inhibitory concentrations and antibiotic susceptibility for Enterobacterales by flow cytometry using reactive oxygen species as a marker
Source: PLoS One. 2025 Sep 4;20(9):e0331217. doi: 10.1371/journal.pone.0331217 (PMC12410812; doi:10.1371/journal.pone.0331217)
Supplement: S3 Table — Acquired flow cytometric data were analyzed using FlowJo. This table shows the matrix used for compensating against the spectral overlap and spillover using the FlowJo software. (DOCX) [file pone.0331217.s004.docx]

**S3 Table:** Compensation matrix used for spectral spill-over of each fluorophore into respective detector used for FCM.

| **Fluorophore\Detector** | **FL1-H: RSG** | **FL3-H: PI** | **FL5-H: SYTO-62** |
| --- | --- | --- | --- |
| **RSG** | 100 | 0.4 | 0 |
| **PI** | 0.5 | 100 | 19.5 |
| **SYTO-62** | 0 | 9.5 | 100 |
