# Supplementary material for: Determining minimal inhibitory concentrations and antibiotic susceptibility for Enterobacterales by flow cytometry using reactive oxygen species as a marker
Source: PLoS One. 2025 Sep 4;20(9):e0331217. doi: 10.1371/journal.pone.0331217 (PMC12410812; doi:10.1371/journal.pone.0331217)
Supplement: S1 File — Details of both the Bland-Altman and Linear Regression analyses (including raw data and equations) were shown in this document. (DOCX) [file pone.0331217.s005.docx]

**1. BLAND-ALTMAN ANALYSIS FOR LIMITS OF AGREEMENT**

| **Parameter** | **Abbreviations/Symbols** | **Value** |
| --- | --- | --- |
| **alpha** | **α** | 0.05 |
| **numbers assessed (n)** | **n** | 24 |
| **degrees of freedom (n-1)** | **n-1** | 23 |
| ***t* value for 23 degrees of freedom  at α = 0.05 (2-tailed test) (*t*)** | ***t*** | 2.0687 |
| **difference mean (đ)** | **đ** | 17.381 |
| **standard deviation (SD)** | **SD** | 42.109 |
| **Upper Limit of Agreement (đ + 1.96×SD)** | **Upper LOA** | 99.913 |
| **Lower Limit of Agreement (đ - 1.96×SD)** | **Lower LOA** | -65.15 |

|  | **value** | **standard error (SE)** | **Confidence (se**t*)** | **lower bounds  [đ (± 1.96×SD) – confidence]** | **upper bounds  [đ (± 1.96×SD) + confidence]** |
| --- | --- | --- | --- | --- | --- |
| **upper LOA** | 99.913 | 14.898 | 30.819 | 69.093 | 130.732 |
| **mean (đ)** | 17.381 | 8.595 | 17.781 | -0.400 | 35.162 |
| **lower LOA** | -65.151 | 14.898 | 30.819 | -95.970 | -34.331 |

| **Mathematical formulas** | **Description** | **Reference** |
| --- | --- | --- |
| $SE=\frac{SD}{\sqrt{n}}$  (Eq.1) | For computing SE for mean | 1. Bland JM, Altman DG. Measuring agreement in method comparison studies. Stat Methods Med Res. 1999 Jun;8(2):135-60. doi: 10.1177/096228029900800204. PMID: 10501650. 2. Giavarina D. Understanding Bland Altman analysis. Biochem Med (Zagreb). 2015 Jun 5;25(2):141-51. doi: 10.11613/BM.2015.015. PMID: 26110027; PMCID: PMC4470095. |
| $SE=\sqrt{\left( \frac{1}{n} \right)+\frac{{1.96}^{2}}{2(n-1)}}\times SD$  (Eq.2) | For computing SE for lower LOA & upper LOA | 1. Bland JM, Altman DG. Measuring agreement in method comparison studies. Stat Methods Med Res. 1999 Jun;8(2):135-60. doi: 10.1177/096228029900800204. PMID: 10501650. |

| Y = β_0_+β_1_(X)+ε | | (Eq. 3) |
| --- | --- | --- |
| Whereby: | | |
| X | mean of the two methods for each sample | |
| Y | (difference between the two methods/mean) × 100% | |
| ε | Residual/Error term | |
| β_0_ | intercept | |
| β_1_ | slope | |

**2. LINEAR REGRESSION ANALYSIS FOR BIASNESS**

Statistical determination for proportional bias:
H₀: Slope (β₁) = 0 (no proportional bias)
H₁: Slope ≠ 0 (there is proportional bias)

SUMMARY OUTPUT FOR LINEAR REGRESSION

| *Regression Statistics* | |
| --- | --- |
| Multiple R | 0.24 |
| R Square | 0.06 |
| Adjusted R Square | 0.014 |
| Standard Error | 42.58 |
| Observations | 23 |

ANOVA (ANALYSIS OF VARIANCE)

|  | *df* | *SS* | *MS* | *F* | *Significance F* |
| --- | --- | --- | --- | --- | --- |
| Regression | 1 | 2385.87 | 2385.87 | 1.316 | 0.2643 |
| Residual | 21 | 38081.21 | 1813.39 |  |  |
| Total | 22 | 40467.08 |  |  |  |

|  | *Coefficients* | *Standard Error* | *t Stat* | *P-value* | *Lower 95%* | *Upper 95%* | *Lower 95.0%* |
| --- | --- | --- | --- | --- | --- | --- | --- |
| Intercept | 45.07 | 25.11 | 1.795 | 0.0870 | -7.138 | 97.29 | -7.138 |
| Slope | -8.851 | 7.716 | -1.147 | 0.264 | -24.90 | 7.196 | -24.90 |

Values obtained from regression analysis

| **Parameter** | **Value** |
| --- | --- |
| alpha | 0.05 |
| Slope (β) | -8.85 |
| *P*-value for slope | 0.264 |
| R² (coefficient of determination) | 0.059 |
| F-statistic *P*-value | 0.264 |
| Intercept | 45.07 |
